# Immune Evasion in Pancreatic Cancer: From Mechanisms to Therapy

**DOI:** 10.3390/cancers10010006

**Published:** 2018-01-03

**Authors:** Neus Martinez-Bosch, Judith Vinaixa, Pilar Navarro

**Affiliations:** 1 Cancer Research Program, Hospital del Mar Medical Research Institute (IMIM), Barcelona 08003, Spain; nmartinez@imim.es (N.M.-B.); jvinaixa@imim.es (J.V.); 2 Institute of Biomedical Research of Barcelona (IIBB-CSIC), Barcelona 08036, Spain

**Keywords:** pancreatic cancer, immune surveillance, galectins, immunotherapy, immune checkpoints, stroma

## Abstract

Pancreatic ductal adenocarcinoma (PDA), the most frequent type of pancreatic cancer, remains one of the most challenging problems for the biomedical and clinical fields, with abysmal survival rates and poor therapy efficiency. Desmoplasia, which is abundant in PDA, can be blamed for much of the mechanisms behind poor drug performance, as it is the main source of the cytokines and chemokines that orchestrate rapid and silent tumor progression to allow tumor cells to be isolated into an extensive fibrotic reaction, which results in inefficient drug delivery. However, since immunotherapy was proclaimed as the breakthrough of the year in 2013, the focus on the stroma of pancreatic cancer has interestingly moved from activated fibroblasts to the immune compartment, trying to understand the immunosuppressive factors that play a part in the strong immune evasion that characterizes PDA. The PDA microenvironment is highly immunosuppressive and is basically composed of T regulatory cells (Tregs), tumor-associated macrophages (TAMs), and myeloid-derived suppressive cells (MDSCs), which block CD8^+^ T-cell duties in tumor recognition and clearance. Interestingly, preclinical data have highlighted the importance of this immune evasion as the source of resistance to single checkpoint immunotherapies and cancer vaccines and point at pathways that inhibit the immune attack as a key to solve the therapy puzzle. Here, we will discuss the molecular mechanisms involved in PDA immune escape as well as the state of the art of the PDA immunotherapy.

## 1. Introduction 

The immune system plays a key role in both the positive and negative regulation of tumor development and progression, and crosstalk between cancer cells and immune cells has been incorporated into the list of major hallmarks of cancer [1]. While immune surveillance [2,3] is the first filter to identify and eliminate aberrant or malignant cells, some tumor cells have developed numerous strategies to avoid recognition by the host immune cells, allowing them to escape from immune control and continue cancer progression. Different mechanisms are involved in tumor immune evasion. First, cancer cells can decrease immune recognition by downregulating antigen presentation pathways, like the major histocompatibility complex (MHC) I proteins, TAP (transporter associated with antigen processing) protein and latent membrane proteins (LMP2 and LMP7) [4,5,6,7,8,9]. Moreover, genetic instability of tumors and constant cell division can result in the loss of tumor antigens recognized by effector T-cells (CD8^+^ or CD4^+^ T-cells). These changes in the immunogenicity of cancer cells leading to immune-resistant clones have been called tumor “immunoediting” [10]. Second, tumor cells and other cells from the tumor microenvironment can promote an immune privilege status by secretion of immunosuppressive cytokines—such as IL-1, IL-6, IL-10, TGFβ, TNFα, or VEGF [11,12,13,14,15,16,17,18]—or modulation of the expression of immunoregulatory molecules to induce T cell anergy or tolerance, such as the immune checkpoints molecules of the B7 family (PD1/PDL1, B7-1, B7-2/CTLA4, B7-H4), A2AR, LAG-3, galectin-9/TIM-3, IDO, and VISTA [19,20,21,22]. Finally, overexpression of STAT3 or BCL-2 by tumor cells increases their resistance to apoptosis and also contributes to immune escape. Altogether, these mechanisms orchestrate an immunosuppressive cancer microenvironment through inhibition of immune cells involved in tumor rejection—CD4^+^/CD8^+^ T lymphocytes and natural killer (NK) cells—together with the recruitment and/or activation of immunosuppressive cells, like CD4^+^CD25^+^FoxP3^+^ T regulatory cells (Tregs), myeloid-derived suppressor cells (MDSCs) and anti-inflammatory M2 macrophages. Although there is considerable knowledge about these immune evasion strategies and how to face them using immunotherapy in several tumor types (such as in melanomas, lung cancer, or lymphomas), little is known about pancreatic cancer. Here, we review the molecular mechanisms involved in the immunosuppressive nature of pancreatic ductal adenocarcinoma (PDA) and the current progress that has been made in immunology-based therapy against this dismal disease.

## 2. PDA Tumor Heterogeneity and Immune Responses

Genomic analyses have revealed the biological complexity and high tumor heterogeneity of PDA. Whole-exome and whole-genome sequencing studies have revealed a high number of somatic copy number alterations and mutations in pancreatic cancer, leading to altered expression of key oncogenes and tumor suppressor genes, such as *KRAS*, *TP53*, *SMAD4*, and *CDKN2A* [23,24,25,26]. More recent studies from the Australian Pancreatic Cancer Genome Initiative/International Cancer Genome Consortium [27] and from The Cancer Genome Atlas (TCGA) network [28] have validated these results and have also added a long list of other less-frequently mutated genes, confirming the complex molecular landscape of pancreatic cancer. These gene expression analyses have allowed PDA patients to be classified according to their molecular signatures into different subtypes that associate with histopathological hallmarks and prognosis, providing new avenues for personalized medicine. The most recent subcategories proposed for PDA include four subtypes: (1) pancreatic progenitor or classical PDA, which express early pancreatic development genes (i.e., *PDX1*, *FOXA2/3, HES1*); (2) squamous or quasimesenchymal tumors, which have increased *TP53* mutations, upregulated *TP63ΔN* and activated TGF-β signaling and MYC pathways and a poor prognosis; (3) aberrantly differentiated endocrine/exocrine tumors, which overexpress genes involved in *KRAS* activation and exocrine (*NR5A*, *RBPJL*) and endocrine (*NEUROD1*, *NKX2-2*) markers; and (4) immunogenic PDA. The last subtype has molecular similarities to classical PDA, but also expresses genes associated to immune phenotypes, such as Toll-like receptors, antigen presentation molecules and genes related to infiltrating B and T cells, both T-cytotoxic (CD8^+^) and Tregs. Moreover, these tumors also show upregulation of the immune checkpoint molecules CTLA4 and PD1, suggesting that the immunogenic PDA subtype may be sensitive to immunotherapy. 

However, most pancreatic tumors do not belong to the immunogenic subtype, and PDA is therefore generally described as a poorly immunogenic tumor. The PDA immunosuppressive microenvironment is mainly composed of Tregs, macrophages and MDSCs, which block the anti-tumoral activity of effector CD4^+^ and CD8^+^ T-cells [29,30,31,32]. These immunosuppressive cells are already present in preneoplastic lesions (PanINs), indicating that they may be key players in tumor initiation [33]. The important role of Tregs in PDA has been shown in a murine model, in which disruption of these cells correlated with tumor growth inhibition [34]. Tumor-associated macrophages (TAMs) also play important functions in pancreatic tumor chronic inflammation, progression and metastasis. TAMs express chemokine (C-C motif) receptor type 2 (CCR2), a chemokine receptor that interacts with chemokine (C-C motif) ligand 2 (CCL2) and exerts a pro-tumoral role mediating tumor proliferation, angiogenesis, and chemotaxis of immune suppressive cells to the tumor stroma [35].

PDA also contributes to immunosuppression by upregulation of negative T cell co-stimulatory molecules [36]. In this regard, PDL1 and PDL2 are overexpressed in PDA patients [37] and correlate with reduced tumor-infiltrating leukocytes (TILs) and worse prognosis [38,39]. Accordingly, downregulation of PDL1 inhibits pancreatic tumor cell proliferation [40]. Furthermore, increased expression of inhibitory molecules on inactivated T cells has been suggested as another way to induce pancreatic cancer immunosuppression. Thus, pancreatic tumors show a high expression of CD40, a cell membrane receptor of the tumor necrosis factor family that modulates immune response, and this overexpression is associated to higher TNM Classification of Malignant Tumours (TNM) staging and metastasis [41]. 

Another reason to explain the PDA immunosuppressive phenotype might be its mutational signature. Indeed, recent cancer mutational analyses have demonstrated high variability among tumors, indicating that those tumors with increased mutation rate, like melanoma or lung, are highly immunogenic while those with low mutation rate, as PDA [23], are poorly antigenic [42,43,44]. Interestingly, very recent data have indicated that neoantigen quality, rather than quantity, may modulate immunogenicity of PDA [45]. This study suggests that the immune response against neoantigens with unique qualities generated during pancreatic tumor evolution, such as neoantigens in mucin 16, can lead to decreased relapse and the best prognosis. Further studies to identify other PDA immunogenic hotspots could be encouraging for the development of neoantigen-targeted strategies for treating checkpoint blockade-resistant patients which, unfortunately, are the vast majority.

## 3. Immune Checkpoints in PDA 

Immune checkpoints refer to several co-stimulatory and inhibitory signals in immune cells that, in physiological conditions, prevent autoimmunity and constrict tissue damage during infections. However, these pathways can be hijacked by tumor cells to achieve immune evasion [46,47]. Among the many different immune checkpoints that regulate T-cell activation, cytotoxic T-lymphocyte-associated antigen 4 (CTLA4) and programmed cell death protein 1 (PD1) are the most well characterized and studied for cancer immunotherapy. 

CTLA4 is expressed by activated T cells and Tregs and binds to its ligands B7-1/CD80 and B7-2/CD86 on the surface of antigen-presenting cells, which leads to downregulation of T helper cells and enhancement of Treg-mediated immune suppression [46,47,48]. Interestingly, a blockade of CTLA4 in preclinical murine cancer models restores effector T-cells and decreases Tregs in melanoma [49,50] and colorectal cancer [51], demonstrating the important role of this molecule in tumor immune evasion. As PDA is characterized for its highly immunosuppressive stroma rich in Tregs, a CTLA4 immune checkpoint emerges as a promising target also for PDA treatment. Indeed, recent data by Bengsch et al. [52] using PDA mouse models have demonstrated that blocking CTLA4 in Tregs induces CD4^+^ T-cell tumor infiltration, suggesting an important role for pancreatic cancer immunotherapy. However, PDA patients enrolled in clinical trials with α-CTLA4 immunotherapy failed to respond to the treatment [53,54,55]. Similar results were obtained in a genetically-engineered PDA murine model (KPC model), in which treatment with α-CTLA4 monoclonal antibodies (mAbs) showed no effects on tumor growth or survival [56]. Nonetheless, T-cell stimulation with agonistic α-CD40 mAbs plus gemcitabine and nab-paclitaxel induced tumor regression and increased survival [56], suggesting that CD40-mediated induction of the T-cell response can overcome PDA resistance to α-CTLA4 immunotherapy (see below). In contrast, other studies using a PDA model after Panc02 subcutaneous injection showed tumor rejection in 50% of mice after α-CTLA4 treatment [57]. These discrepancies in α-CTLA4 therapy efficacy can be a consequence of the origin of Panc02 cells, which are derived from a mouse tumor generated by carcinogen induction [58] and, therefore, are probably hypermutated and display higher antigenicity than KPC tumors (and most human PDAs, see Section 2 (PDA Tumor Heterogeneity and Immune Responses).

Regarding the PD1/PDL1 immune checkpoint, PDL1 overexpression has been reported in PDA tumors, both in tumor cells and in the scarce immune infiltrates [38,56], and increased PDL1 expression is associated with poor survival [38]. Controversial data regarding the correlation of PDL1 expression and presence of infiltrating CD8^+^ T cells [38] have been reported [38,56]. PD1 has been found to be expressed in about half of pancreatic tumor cytotoxic infiltrates [59]. Overexpression of PDL1 is also found in the KPC mouse model of PDA, for which moderate staining was observed in around 40% of tumor cells as well as in stromal dendritic cells (DCs) and macrophages, which express higher PDL1 levels than their counterparts in the spleen. Moreover, tumor-infiltrating T-lymphocytes, including Tregs and few CD4^+^ and CD8^+^ T cells, also express PD1 at higher levels than that of the spleen populations [56]. These results point to a role of the PD1/PDL1 immune checkpoint in PDA immune evasion. Similar to α-CTLA4 therapy, Pan02 tumors provide objective responses to α-PD1 or α-PDL1 therapies [38,60]. However, KPC tumors are refractory to α-PD1 or α-PDL1 mAbs, either alone or in combination with α-CTLA4 therapy; this resistance could be reverted, however, by combined treatment with α-CD40 and chemotherapy [56]. Importantly, Winograd et al. demonstrated that this combined regimen not only induces tumor rejection in a CD8^+^ T-cell-dependent manner, but also increases the capability of mice to reject subsequent tumor insults, suggesting that an anti-tumor immune memory with high therapeutic potential is generated. Indeed ongoing clinical trials in patients with α-CD40 are giving promising results [61] (see below). 

## 4. Role of Galectins in Immune Evasion in PDA

Among the different mechanisms involved in immune evasion, galectins—a family of proteins with high affinity for β-galactoside residues—have recently emerged as one of the key players in the regulation of lymphoid and myeloid cells in cancer. This family comprises 15 members, which share a consensus carbohydrate recognition domain (CRD) responsible for their glycan binding [62,63,64]. Some galectins contain only one CRD and are active as monomers (galectin [Gal]-5, -7, and -10) or dimers (Gal-1, -2, -11, -13, -14, and -15), while Gal-4, -6, -8, -9, and -12 contain two CRD linked by a short peptide. Gal-3 is the only member that contains one CRD and a non-lectin domain, which allows its oligomerization [63,65]. Although galectins can act both via sugar-dependent and sugar-independent interactions, most of their biological functions are via interactions with glycosylated proteins on the cell surface or in the extracellular matrix, playing key roles in cell-cell adhesion, migration and signaling. Galectins can be localized in different cell compartments [66] and also secreted extracellularly through a non-classical secretory pathway [67]. 

Importantly, altered expression of different members of the galectin family has been reported in several cancer types. In particular, Gal-1 overexpression is frequently associated with poor prognosis of many tumors [68], while Gal-3 and Gal-9 expression are tumor type–dependent [66]. Overexpression of galectins in cancer induce pro-tumoral functions like proliferation, epithelial to mesenchymal transition, angiogenesis and, remarkably, tumor immune evasion [69,70,71,72,73]. 

The role of galectins in the regulation of immune system has been widely studied. Gal-1 can recognize a variety of glycoproteins on the T-cell surface, inhibiting transendothelial T-cell migration, T-cell activation and, importantly, promoting apoptosis of activated Th1 and Th17 CD8^+^ T cells, thereby tilting the immune balance towards a Th2 profile [74,75]. Additionally, Gal-1 can induce Treg and DC differentiation, impair NK cell recruitment to the tumor, induce M2 macrophage polarization and promote expansion of MDSCs and γδ-T cells [76,77]. Altogether, these modulatory functions on different immune cell populations lead to a key role for Gal-1 in immune evasion, both in physiology during self-recognition and in pathology during cancer progression. Gal-1’s contribution to tumor immunosuppression has also been reported in PDA. Pancreatic stellate cells (PSC) are responsible for Gal-1 secretion and overexpression in the tumor microenvironment. In this context, extracellular Gal-1 secreted by PSC reduces viability of both CD4^+^ and CD8^+^ infiltrating activated T-cells [78]. Moreover, a high level of Gal-1 favors a significant anti-tumor Th2 cytokine secretion and a drastically decrease of Th1 cytokine production [78]. Importantly, Gal-1 is highly expressed in the stroma of human and murine pancreatic tumors, where it drives tumor progression through stroma activation, induced angiogenesis tumor cell proliferation and acinar-to-ductal metaplasia [79]. Of note, when genetically inhibiting Gal-1, tumors show a significantly enhanced number of T-cell infiltrates and neutrophils [79], suggesting that galectin-1 may be key in driving immune evasion in PDA [80,81]. 

Gal-3 regulates immune system responses that act as a chemoattractant for monocytes and macrophages [82], impairing NK anti-tumor function. It also controls expansion of DCs [77] and is able to modulate T-cell responses through apoptosis, T-cell receptor downregulation and crosslinking, consequently inhibiting T-cell activation [83]. Gal-3 also promotes reduced immune response by decreasing IL-5 production and blocking B lymphocyte differentiation [84]. Interestingly, in PDA, treating tumor-infiltrating CD8^+^ T-cells with α-Gal-3 mAb boosts their activation by increased IFNγ secretion. Upon ex vivo restimulation, the polysaccharide GCS-100, which is in clinical development, can detach Gal-3 from TILs. This favors activation of both CD8^+^ and CD4^+^ T cells and secretion of anti-tumor cytokines. Importantly, in vaccinated tumor-bearing mice, GCS-100 injections result in tumor regression [85]. Of note, Gal-3 binding to LAG-3 induces CD8^+^ T cell suppression in vitro and Gal-3 KO mice show increased T effector functions upon GM-CSF vaccine administration [86]. Indeed, phase I clinical trials (with still inconclusive results) have already been designed with Gal-3 inhibitors in combination with peptide vaccines, as well as with the immune checkpoint inhibitors ipilimumab (α-CTLA4) and pembrolizumab (α-PD1), in metastatic melanoma. Importantly, Gal-3 has already been added to the list of “new generation” checkpoints in immunotherapy [47]. 

Finally, Gal-9 also has crucial roles in controlling immune regulatory circuits, both in immune cell homeostasis and during cancer immune surveillance. Gal-9 can induce Treg cell differentiation and promote expansion of immunosuppressive MDSCs. Importantly, it specifically triggers apoptosis by interacting with TIM-3 in Th1 and CD8^+^ T cells [77,87], which has also placed Gal-9 in the “new generation” checkpoint list [47,88]. Interestingly, Gal-9 has the ability to modulate immune responses in PDA [89]. Gal-9 is a ligand for dectin-1, which is an innate immune receptor highly expressed in macrophages in PDA. The dectin-1/Gal-9 axis plays a key role in promoting differentiation of TAMs to a M2-like phenotype. Importantly, Gal-9 inhibition restores intratumoral T-cell infiltrates in PDA, but this function is impaired in the context of a dectin-1 deletion, suggesting that the dectin-1/Gal-9 interaction is key in reprogramming CD4^+^ and CD8^+^ T-cells for regulating tumor progression; thus, highlighting these players as targets in immunotherapy [89]. 

## 5. Other Molecular Mechanisms of Immune Escape in PDA 

Preclinical evaluation of checkpoint inhibitors in PDA therapy has been considered; however, as PDA is a non-immunogenic tumor, monotherapy blocking of the PD1/PDL1 axis or CTLA4 have not been successful [56,90]. Nevertheless, strategies stimulating the immune system in parallel with other treatments have shed some light on the field and present promising outcomes. 

An α-CD40 antibody plus chemotherapy alters the phenotype of TILs, and, in combination with α-PD1 or α-CTLA4, or both, leads to regression of established tumors due to reduced number of Tregs and enhanced CD8:Treg ratio [56,91]. Interestingly, Feig et al. reported that depletion of FAP^+^ cells in the tumor hampered tumor growth, due to increased infiltrating CD4^+^ and CD8^+^ T-cells, and synergized with checkpoint monotherapies [90]. C-X-C motif chemokine ligand 12 (CXCL12) was found to be the chemokine responsible for recruiting effector T cells, and targeting its receptor (CXCR4) resulted in reduced pancreatic tumor growth in a T-cell-dependent manner. In combination with α-PDL1, the number of proliferating cancer cells in tumors are greatly reduced [90]. Additionally, in a more recent report [92], CXCR2 inhibitors (which increase the amount of infiltrating CD3^+^ T-cells in KPC mice) were administered in a priming phase to enhance pancreatic T-cell infiltration, and animals were subsequently treated with either a combination of CXCR2 inhibitors and α-PD1 or directly with α-PD1 as a control. Immune checkpoint blockade proved to be efficient when targeting CXCR2 in parallel, significantly increasing animal survival by reducing the proliferation tumor rates as well as enhancing the proportion of both CD4^+^ and CD8^+^ T cells while diminishing Tregs. IL-6 blockade in combination with α-PDL1 also increased T-cell infiltration and synergistic outcomes in immunocompetent mouse models [93]. Ruxolitinib (a JAK-Stat inhibitor), which inhibits systemic inflammation in the stroma of pancreatic cancer, also enhances cytotoxic T lymphocyte infiltration and boosts α-PD1 therapy in an orthotopic mouse model of PDA [94]. 

Multiple additional different designs have been proposed in preclinical studies to target the PDA immunosuppressive microenvironment and to increase immunotherapy efficiency, such as combinations with chemotherapy, radiation, therapeutic vaccines, or even several of these together [95]. For instance, addition of α-PDL1 to high radiotherapy doses improved responses in KPC and Pan02 allografts, by shifting the balance towards increased CD8^+^ T cells at the expense of reducing CD11b^+^Gr1^+^ myeloid infiltrations [96]. The combination of checkpoint inhibitors with therapeutic vaccines to prime the microenvironment with effector T cells before repressing the inhibitory signals [97] has also shown impressive results. For example, GVAX together with α-PD1 therapy improves survival of tumor-bearing mice [98]. More sophisticated mechanisms have also provided alternatives to increase immunotherapy efficiency, such as targeting the MLL1-H3K4me3 epigenetic axis [59] or using antiangiogenic therapy to form intratumoral HEVs (high endothelial venules) to facilitate CTL tumor infiltration [99]. 

## 6. PDA and Immunotherapy 

Pancreatic cancer clearly remains in dire needs of efficient treatment options. Considering that the current chemotherapy regimens have only led to extremely low levels of improvement in survival rates, and in light of the data showing that the immunosuppressive microenvironment is a feasible source of resistance, immunotherapy in clinical trials has raised high expectations. However, results from the initial studies with checkpoint inhibitors have not met expectations, and more sophisticated combinations are now being contemplated. 

**Figure 1:**
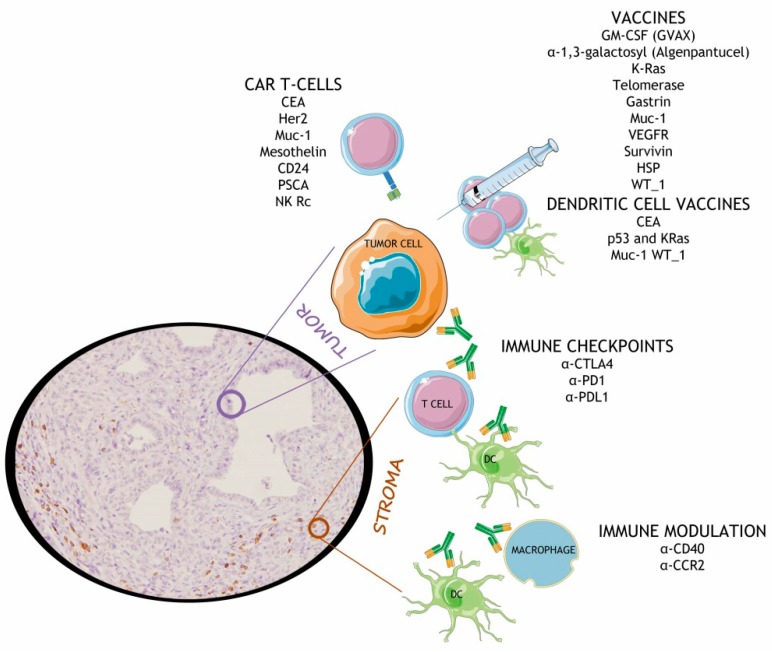
Immunotherapy strategies that have been considered in PDA therapy. Targeting immune checkpoints, the use of vaccines, CAR T-cells or immune modulating molecules have been included in pancreatic cancer clinical trials. CEA: carcinoembryonic antigen; Her2: human epidermal growth factor receptor 2; Muc-1: mucin-1; CD24: Cluster of differentiation 24; PSCA: prostate stem cell antigen; NK Rc: natural killer receptor; GM-CSF: granulocyte-macrophage colony-stimulating factor; VEGFR: vascular endothelial growth factor; HSP: heat shock protein; WT-1: Wilms tumor 1; CTLA4: cytotoxic T lymphocyte–associated protein 4; PD1: programmed cell death protein 1; PDL1: programmed death ligand-1; CD40: cluster of differentiation 40; CCR2: C-C motif chemokine receptor 2.

The first pathways to be tested in clinical trials concerned checkpoint inhibitions (Figure 1). α-CTLA4 ipilimumab was assessed in a phase II clinical trial [54] in patients with advanced disease. RECIST (response evaluation criteria in solid tumors) reported an overall lack of response, although the condition of one patient (of 27) did improve due to the treatment. Several clinical trials with ipilimumab have been organized, which combine it with chemotherapy and/or stimulators of the immune response, such as GVAX; these have achieved mild improvements in overall survival (OS) and one-year OS [55]. Most of these combinatory clinical trials have not yet published their results. A phase I dose escalation of another α-CTLA4 (tremelimumab) has been also performed in patients with metastatic pancreatic cancer [100], opening the door to further advanced clinical trials to evaluate its safety and efficacy, alone or in combination with radiation and other immune checkpoint inhibitors. The first results on treating pancreatic cancer with α-PD1 pembrolizumab have just been published. The PembroPlus phase Ib study [101] studied the safety of combinations of α-PD1 and different chemotherapy agents, although conclusions about efficiency require further data. A phase Ib/II study of gemcitabine, nab-paclitaxel and pembrolizumab in 17 metastatic PDA patients [102] was recently presented without meeting its primary endpoint. Twenty clinical trials with pembrolizumab in pancreatic cancer are active at the moment. BMS-956559 α-PDL1 achieved good performance in a phase I study with different solid tumors, although no objective responses were observed in pancreatic cancer patients [53]. Eleven clinical trials are running with α-PDL1 durvalumab in pancreatic cancer, in different combinations. Although CTLA4 and PD1/PDL1 are the most well-studied immune checkpoints, other molecules of this type, such as LAG-3, TIM-3, and A2AR, also function to shut down the immune response and are also being considered for immunotherapy in clinical trials [103].

Therapeutic cancer vaccines using cancer cell antigens mixed with agents that stimulate patient immune responses have shown impressive results in several tumors and are also in the spotlight in pancreatic cancer clinical trials (Figure 1). For instance, GVAX (a GM-CSF vaccine) presented favorable results in a phase I study [104] and phase II studies in combination with cyclophosphamide (to reduce Tregs) [105] or chemoradiation [106], uncovering an interesting prognostic factor through the correlation between induction of mesothelin-specific T-cell responses with improved overall survival. Additionally, a phase II clinical trial that used GVAX in combination with CRS-207 (a live-attenuated strain of a *Listeria monocytogenes*–encoded mesothelin), to enhance both the innate and adaptive immunity, showed improved overall survival [107]. Positive results for the vaccine Algenpantucel-L have also been published in a phase II study [108] in combination with gemcitabine or fluorouracil, leading to ongoing promising phase III clinical trials (IMPRESS and PILLAR trials). Clinical trials with peptide vaccines instead of whole-cell vaccines have also been organized. A phase I/II trial with K-Ras peptides in combination with GM-CSF was performed, with partial positive responses [109]. Further evidence for the potential of using a Ras vaccine in pancreatic cancer was demonstrated by a subsequent trials [110,111,112], with encouraging results and impressive 10-year follow-ups. Interestingly, the GI-4000 vaccine (which triggers an immune response against mutated Ras) has also drawn clinician’s attention [113]. The telomerase peptide vaccine (GV1001) showed successful results in a phase I/II trial [114], although phase III clinical trials with chemotherapy regimens have not been able to show a survival advantage [115]. Although different results have been achieved depending on combinations, other peptide-based vaccines have also led to pancreatic cancer patients inducing an antibody response but without achieving impressive results; these include the MUC1 vaccines [116], anti-VEGFR vaccines [117,118], survivin [119], anti-gastrin [120], anti-heat shock protein vaccine [121], and anti-WT-1 [122]. Dendritic cell vaccines are also under investigation in pancreatic cancer clinical trials, highlighting carcinoembryonic antigen (CEA)-loaded DCs [123], mutated p53 and K-Ras loaded DCs [124], MUC1 [125], and WT-1 pulsed DCS [126]. Interestingly, very recently publications have shown that therapy with DCs together with CIK (cytokine-induced killer cells) and chemotherapy associates with increased immune responses, resulting in favorable progression free survival and OS [127]. 

Another strategy that is revolutionizing cancer immunotherapy is adoptive T-cell therapy, in which autologous or allogenic tumor-specific cytotoxic T cells are infused into cancer patients with the goal of recognizing, targeting, and destroying tumor cells. The most universal approach is to using T cells that are genetically modified to express chimeric antigen receptors (CARs) specific tumor-associated antigens (such as CEA, EGFR, Her2, MUC1, mesothelin, CD24, and PSCA). This strategy has performed relatively well in pancreatic cancer preclinical studies [128,129,130,131,132,133,134]. However, taking the plunge to the clinic has not proven as successful, probably due to the tumor immunosuppressive microenvironment, and neoadjuvant chemoradiation and combinations with costimulators are now being explored (Figure 1). Interestingly, strategies have been designed to enhance CAR T-cell therapy efficiency, such as reducing tumor bulk before adoptive cell therapy, optimizing the Th2-to-Th1 ratio of infused CAR T-cells, pre-conditioning to deplete Tregs (by inhibiting CTLA4, CD-25 targeting, TNFα agonism or combining with rosiglitazone), increasing CD8^+^ TILS (with metformin or by inhibiting TGF-β), increasing macrophage activation (by targeting CSF1, CCR2 or Bruton tyrosine kinase (BTK)) and even co-expressing albumin on the surface of CAR T-cells (to enhance accumulation in the tumor) [134]. 

Considering that the strong immunosuppressive barrier is a handicap in pancreatic cancer therapy, approaches with immune-modulating agents are also being considered in clinical trials (Figure 1). A CD40 agonist antibody was combined with gemcitabine for treating 22 advanced PDA patients [61]; this achieved promising results and opens the door to a couple more clinical trials with CD40 agonist and chemotherapy, with results yet to be published. In a similar context, inhibition of CCR2 to target TAMs and monocytes in combination with folfirinox led to either stabilization of disease or partial response in all of the patients who could be evaluated according to RECIST [135]. 

An interesting meta-analysis gathering all clinical trials with immunotherapies in pancreatic cancer [136] found out that this strategy indeed significantly increases the 3-, 6-, 12-month and 3-year OS of patients. Importantly, immunotherapy significantly increases the immune response of patients with PDA and reduced CA19-9 levels.

In conclusion, a large effort to fit immunotherapy into pancreatic cancer therapies is being made by companies and clinicians, but it is still far from being the panacea. The strong immunosuppressive PDA microenvironment becomes a double-edged sword: on one hand, it makes immunotherapy a reasonable option as a therapy, but on the other, it is, per se, a stumbling block rather than a stepping stone that hinders very much its own efficiency. More sophisticated combinations and personalized patient stratification will be necessary to achieve favorable outcomes and implement this as practical therapy in the clinics. 

## 7. Concluding Remarks

Pancreatic cancer has a complex molecular landscape that handicaps both research advances and therapy efficiency. The strong immunosuppressive microenvironment in PDA, which mainly consists of Tregs, macrophages, and MDSCs, blocks T effector cells, leading to immune evasion and rapid tumor progression. Indeed, checkpoint monotherapies have failed in clinical trials due to this strong immunological barrier. The main actor driving this process to bypass immune surveillance is a tangled combination of chemokines and receptor-ligand signaling pathways, both in the tumor and the stroma, and understanding this is critical to target the key regulators and allow immunotherapy to be efficient. In this direction, basic research and preclinical trials with immunocompetent mice have shed some light on the exact molecular mechanisms involved but are also raising concerns about the complexity of the issue and the feasibility of deriving a combination that is suitable for a high percentage of patients. Indeed, of the roughly a hundred clinical trials with immunotherapy in pancreatic cancer that have been designed, none have achieved impressive results, although clearly some of the patients have had with objective responses and may benefit from the therapies boosting their immune systems. In this regard, new ultrasequencing data from cancer international consortiums have allowed human pancreatic tumors to be classified according to their molecular signature; this has unveiled a new immunogenic subtype that might be more sensitive to immunotherapy. Once again, it seems that we are underestimating the heterogeneity of cancer disease, and that although economic issues cannot be undervalued, the real solution will need to approach the issue through personalized medicine.
